# Educator's Blueprint: Seven Strategies for Turning Peer Review Into Scholarly Growth

**DOI:** 10.1002/aet2.70265

**Published:** 2026-10-06

**Authors:** Dayle Davenport, Wendy Coates, Adaira Landry, Jaime Jordan, Michael Gottlieb

**Affiliations:** ^1^ Department of Emergency Medicine University of Chicago Chicago Illinois USA; ^2^ Department of Emergency Medicine University of California, Los Angeles, David Geffen School of Medicine Los Angeles California USA; ^3^ Department of Emergency Medicine, Brigham and Women's Hospital Harvard Medical School Boston Massachusetts USA; ^4^ Department of Emergency Medicine Oregon Health & Science University Portland Oregon USA; ^5^ Department of Emergency Medicine Rush University Medical Center Chicago Illinois USA

## Abstract

Peer review is central to scholarly publishing and academic advancement in medicine. However, many authors, particularly residents and early‐career faculty, receive limited formal training in navigating the peer review process. This Educator's Blueprint provides seven strategies to optimize journal publication success by improving submission quality and reframing rejection as part of the growth process, while also discussing how to build upon the experience to enhance long‐term scholarly productivity and impact. These include identifying potential journals, tailoring the manuscript to the target journal, crafting a compelling cover letter, responding to reviewer comments, turning rejection into progress, disseminating findings to broaden reach, and leveraging the experience to build a cohesive scholarly portfolio.

## 1 Introduction

Peer review ensures the quality, rigor, and credibility of published research [1, 2] and is essential for authors seeking to disseminate scholarly work and advance professionally [3, 4, 5, 6, 7]. It strengthens scientific discourse by providing expert evaluation that refines methodology, clarifies interpretation, and improves the quality of scholarly work [1, 5].

Despite its importance, the peer review process can be intimidating and is often poorly understood, particularly by early‐career authors [8, 9], who frequently perceive rejection as a personal failure rather than a routine component of publishing [10, 11]. These misconceptions are compounded by limited and inconsistent training on manuscript submission [2, 12]. Existing resources primarily emphasize research methodology or manuscript preparation. Few offer practical guidance for navigating submission, peer review, revision, and rejection. Carmelli et al. recently provided a broad, practical guide to the submission‐to‐publication process for medical educators [13]. Building on this work, our manuscript presents actionable strategies, with particular attention to reframing rejection as a developmental opportunity and leveraging the peer review experience to build a cohesive, longitudinal scholarly portfolio—areas that remain underdeveloped in the existing literature.

This Educator's Blueprint uses peer review as a starting point to outline seven concrete strategies spanning revision, rejection, dissemination, and portfolio‐building for sustained scholarly growth (Figure 1).

**FIGURE 1:**
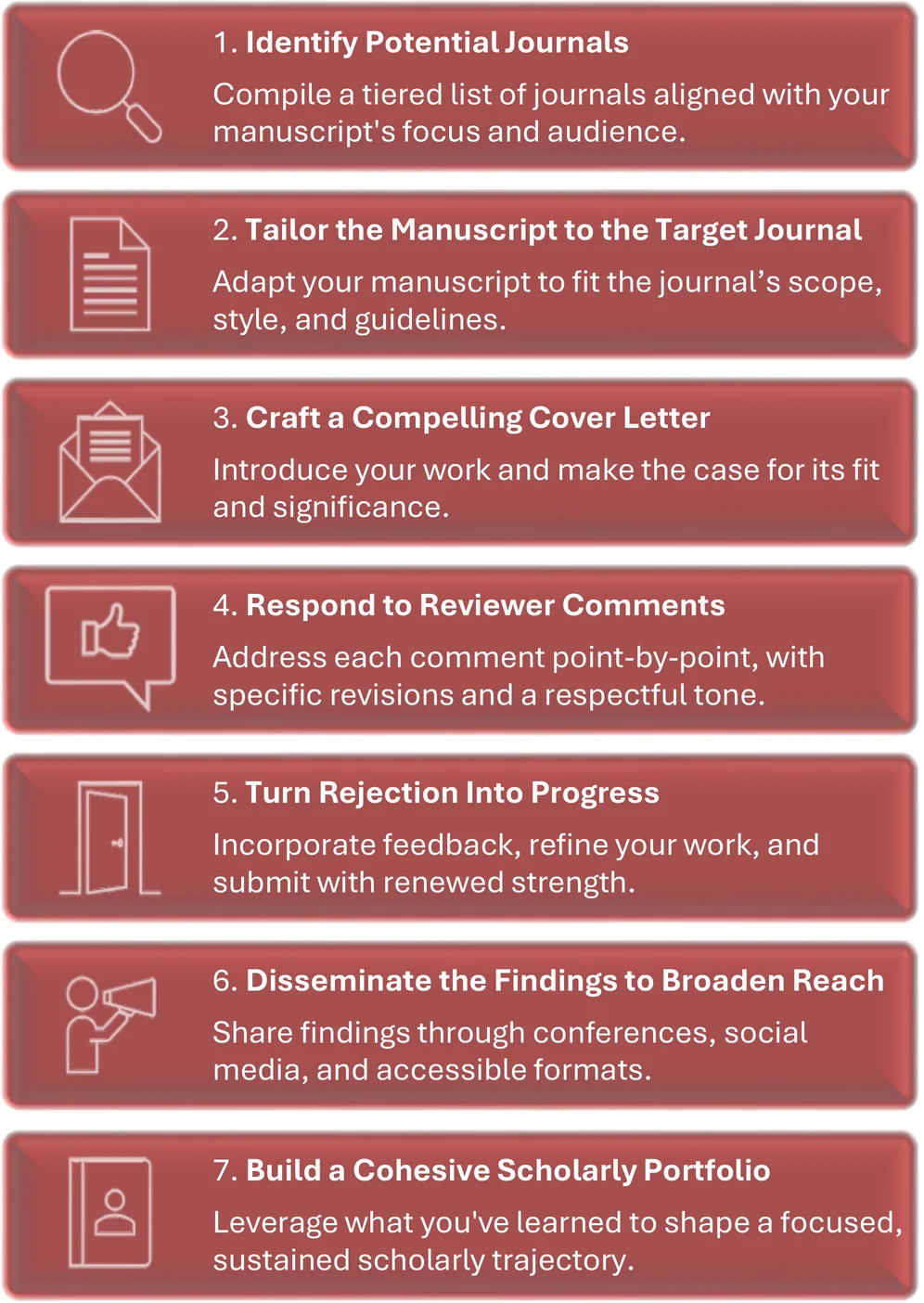
Seven strategies for turning peer review into scholarly growth.

## 2 Strategies

### 2.1 Identify Potential Journals

Rather than selecting a single target journal immediately, authors and mentors should compile a list of suitable journals, prioritizing alignment between the article's focus and the journal's aims [11]. Tailoring the manuscript to a target journal is far easier during development, since decisions regarding manuscript structure, framing, and reporting guidelines can be incorporated while the manuscript is still being drafted. A tiered journal list also preemptively establishes a sequential plan for resubmission, keeping the manuscript moving forward if it is rejected.

Nuanced differences may influence the final selection, including acceptance rate, journal reach and visibility (impact factor), audience, publishing costs to the author (article processing charges [APCs]), open access vs. paywalled content, and publication timelines. Special issues or calls for papers targeting a specific topic can offer a good fit. Some universities cover APCs, and additional discounts may apply based on the corresponding author's country or institution. Arrangements change over time, so authors should confirm current coverage.

The Journal/Author Name Estimator (JANE) database uses a manuscript's title and abstract to suggest journals publishing similar work and ranks the likelihood of a good fit [14] (Figure S1). However, its PubMed‐based indexing may underrepresent newer or non‐indexed educational journals, so its suggestions should be supplemented with other approaches. The Association of American Medical Colleges Group on Educational Affairs maintains a curated list of journals publishing educational scholarship [15], and large language model (LLM) tools can help compare journals on audience, reach, impact factor, turnaround time for decisions, acceptance rates, and APCs.

Many authors aim for journals with the highest‐impact factor [16, 17, 18], but a mismatch in rigor or content reduces the odds of success. A lower‐impact journal with a well‐aligned readership may ultimately have more influence than a broader, higher‐impact one. Some manuscripts must be submitted first to a sponsoring organization's own journal, so authors should confirm this in advance.

### 2.2 Tailor the Manuscript to the Target Journal

Authors should align their manuscript to the guidelines and audience of that specific publication [3, 11] and review the journal's published articles to confirm their manuscript fits its scope. The introduction and discussion sections should emphasize practical implications and relevance to the journal's readership. A well‐tailored manuscript signals to editors that the submission is a purposeful contribution to their readers [19].

Prior to submission, authors should also review the journal's author guidelines, which specify article categories and category‐specific requirements (e.g., word count, format, author limits) [3, 11], as well as reference formatting, figure and table formatting, and applicable reporting and trial registration standards. Failing to follow these requirements is a common and preventable reason for rejection or submissions being returned for edits. A checklist approach helps ensure nothing is overlooked [11, 20]. Artificial intelligence tools, such as LLMs, can also help review journal guidelines and flag deviations before submission [21]. Because journal policies on AI usage range from disclosure to prohibition, authors should consult and follow the target journal's specific guidelines.

### 2.3 Craft a Compelling Cover Letter

The cover letter is often the editor's first introduction to a manuscript and plays an important role in determining whether the manuscript is rejected immediately or advanced to external peer review [22, 23]. An effective letter should be addressed to the editor and include the following:

− Journal name, submission date, manuscript title, and article type
− Invited manuscript or submission for special issue
− Authorship: indicate corresponding author; contact information, author affiliations, and relevant expertise
− Work is original and not under consideration elsewhere
− Ethical disclosures, conflict of interest, and funding statements, permissions for reused materials, and disclosure of use of artificial intelligence
− Explanation of how the work advances current knowledge and aligns with the journal's aims and scope

The letter should remain concise, professional, and transparent, ending with a brief acknowledgment thanking the editor for consideration of the submission. A polished cover letter helps establish credibility and demonstrates thoughtful alignment between the manuscript and the journal's mission [22, 23].

### 2.4 Respond to Reviewer Comments

After undergoing peer review, some manuscripts are eligible for reconsideration after revision. Expert peer reviewers, staff editors, and decision editors will provide comments to improve and clarify the manuscript. Revisions should be framed as an opportunity for iterative improvement, rather than as a personal judgment. Feedback can provoke strong emotional reactions, particularly when comments feel harsh or substantial revisions are requested. Authors may benefit from a brief pause before responding to let initial frustrations subside, while still meeting the revision deadline [8, 10]. If authors cannot adhere to the revision deadline (e.g., travel, illness), contacting the editorial team to request an extension is reasonable.

Responses should address each of the editor and reviewer comments in a point‐by‐point format that reproduces the reviewer's comment verbatim followed by the authors' reply. The first author usually drafts the initial response, with co‐authors addressing specific comments as needed. This can be presented in sequential lines or a grid, with responses distinguished by bold or a different color to help editors and reviewers track revisions easily. Replies should specify exactly what was changed, with page and line references. Quoted excerpts of new or modified text can be helpful. Tone is also important. Authors should express appreciation, remain respectful even when disagreeing, and avoid defensive phrasing. Vague responses like “This has been addressed” should be replaced with concrete descriptions of the revisions, such as “We have noted the single‐center design as a limitation to generalizability (Page 4, Line 170).”

Several situations require additional care. When reviewers offer conflicting recommendations, authors should select the most scientifically defensible path, explain their reasoning objectively, and invite the editor to adjudicate if needed. Contacting the editor before resubmission can help when comments are unclear or conflicting. Comments that appear to reflect a misunderstanding should be interpreted as evidence that the manuscript text was ambiguous and requires clarification [24]. If authors disagree with a reviewer's requested revision, they should provide a clear, evidence‐based rationale (e.g., “We respectfully disagree that…”). Disagreements among co‐authors regarding how to address specific recommendations should be resolved before resubmission, with the first and/or senior author ensuring the final response reflects a unified position.

### 2.5 Turn Rejection Into Progress

While rejection is a common and expected part of academic publishing, it can still feel discouraging, especially for those early in their research career. Rejection should not be interpreted as a definitive judgment on the work's merit or a personal failure. Many factors beyond the manuscript's scientific rigor contribute to editorial decisions, including audience fit, publication space, and differing reviewer perspectives [25, 26, 27]. Discussions with mentors and/or senior authors can help normalize the experience and support resilience, scholarly momentum, and a view of rejection as a routine part of an academic career [5].

Viewing the path to publication as an iterative process rather than a single pass‐fail event can help authors maintain perspective and remain engaged in scholarly work [25]. Following rejection, authors should carefully consider reviewer and editor comments to determine next steps. In most cases, this means incorporating reviewer feedback and moving to the next journal on their tiered list. Occasionally, the same journal may accept the manuscript in a different category. Some journals offer a formal appeal process for decisions reflecting a factual error or misinterpretation, though this route is uncommon and requires substantive grounds.

### 2.6 Disseminate the Findings to Broaden Reach

Once the study is published, authors should actively disseminate the findings beyond the journal's table of contents. Translate the findings into formats accessible to broader audiences. This can include conference didactics as well as leveraging media (e.g., posts/threads, blogs, podcasts) targeted to different audiences. Images and infographics can further enhance knowledge dissemination and increase the likelihood that your audience will read the manuscript [28, 29]. Given the growing volume of publications in medical education and competition for attention, dissemination is essential to ensure the findings are utilized in practice [30, 31, 32, 33].

### 2.7 Leverage the Experience: Building a Cohesive Scholarly Portfolio

The process of designing a study, navigating peer review, and publishing a manuscript develops knowledge and skills that extend well beyond a single paper [34]. Authors should leverage the experience and knowledge gained to cultivate a cohesive scholarly focus and portfolio. A natural next step is a follow‐up study addressing limitations identified during the research or review process, since reviewers' critiques often illuminate promising directions that were not pursued initially. Methodological expertise developed during the project can be applied to new research questions and shared with collaborators and trainees. For example, authors who use the Delphi approach to develop core competencies in cardiac ultrasound could apply a similar model to other ultrasound applications [35]. Literature reviewed extensively for one project can similarly be repurposed into a review paper, didactic, or podcast. Authors should also revisit their notes for related gaps identified during their initial literature review. Publishing repeatedly within a focused area helps authors establish their niche and advance the broader scholarly conversation.

## 3 Conclusion

In this article, we described seven strategies for navigating the peer review process, especially for residents and early‐career faculty with limited formal training. By approaching journal selection, manuscript tailoring, reviewer responses, and rejection strategically, authors can improve their odds of publication while building resilience. Dissemination and reflection on lessons learned extend that value further, turning each submission into a building block for a lasting scholarly portfolio.

## Author Contributions

**Dayle Davenport:** writing – review and editing, writing – original draft. **Wendy Coates:** writing – original draft, writing – review and editing. **Adaira Landry:** writing – original draft, writing – review and editing. **Jaime Jordan:** writing – original draft, writing – review and editing. **Michael Gottlieb:** conceptualization, writing – original draft, writing – review and editing.

## Conflicts of Interest

W.C., J.J., M.G. are on the Editorial Board for *AEM Education & Training*. They were not involved in the peer review selection or decision to publish this manuscript. The remaining authors declare no conflicts of interest.

## Supporting information


**Figure S1:** (a) Suggested journals for the current article using title only. (b) Suggested journals for the current article using the abstract.

## Data Availability

The authors have nothing to report.

## References

[1] J. Kelly , T. Sadeghieh , and K. Adeli , “Peer Review in Scientific Publications: Benefits, Critiques, and a Survival Guide,” EJIFCC 25 (2014): 227–243. PMC4975196 27683470

[2] J. N. Love , C. Merritt , J. S. Ilgen , et al., “A Collaborative Approach to Mentored Peer Reviews Sponsored by the Council of Residency Directors in Emergency Medicine,” Western Journal of Emergency Medicine 25, no. 1 (2024): 111–116. 10.5811/westjem.61488 PMC10777179 38205992

[3] V. V. Bhende , T. S. Sharma , M. Krishnakumar , A. S. Ramaswamy , K. Bilgi , and S. P. Mankad , “Decoding the Rejection Code: Understanding Why Articles Get Axed,” Cureus 16, no. 3 (2024): e56920. 10.7759/cureus.56920 PMC10963210 38533321

[4] M. E. L. Brown , T. M. Chan , M. Gottlieb , G. A. Patino , and L. W. Roberts , “Appreciation for Peer Review and Peer Reviewers in Academic Medicine,” Academic Medicine 98, no. 1 (2023): 1–2. 10.1097/ACM.0000000000005025 40318150

[5] M. Gottlieb , E. Dehon , J. Jordan , et al., “Getting Published in Medical Education: Overcoming Barriers to Scholarly Production,” Western Journal of Emergency Medicine 19, no. 1 (2018): 1–6. 10.5811/westjem.2017.11.35253 PMC5785173 29383048

[6] K. Gore , P. Dyne , M. T. Fitch , A. Mattu , M. T. Pillow , and M. Gottlieb , “Educator's Blueprint: A Guide for Clinician‐Educators to Achieve Promotion in Emergency Medicine,” AEM Education and Training 7, no. 3 (2023): e10883. 10.1002/aet2.10883 PMC10227173 37261218

[7] M. Gottlieb , T. M. Chan , L. M. Yarris , J. A. Linden , and W. C. Coates , “Promotion and Tenure Letters: A Guide for Faculty,” AEM Education and Training 6, no. 3 (2022): e10759. 10.1002/aet2.10759 PMC9178369 35707393

[8] N. Garg and M. Gottlieb , “Resident Research Tips: Surviving and Thriving Within the Peer Review Process,” Annals of Emergency Medicine 71, no. 2 (2018): 247–251. 10.1016/j.annemergmed.2017.09.015 29089170

[9] M. Gottlieb , S. Lotfipour , L. Murphy , C. K. Kraus , J. R. Langabeer, 2nd , and M. I. Langdorf , “Scholarship in Emergency Medicine: A Primer for Junior Academics. Part I: Writing and Publishing,” Western Journal of Emergency Medicine 19, no. 6 (2018): 996–1002. 10.5811/westjem.2018.39283 PMC6225948 30429932

[10] R. Weinstein , “Surviving Peer Review,” Journal of Clinical Apheresis 35, no. 5 (2020): 469–476. 10.1002/jca.21822 32770560

[11] M. S. Tullu and S. Karande , “Success in Publishing: Selecting an Appropriate Journal and Braving the Peer‐Review Process,” Journal of Postgraduate Medicine 64, no. 1 (2018): 1–4. 10.4103/jpgm.JPGM_618_17 PMC5820808 29386410

[12] J. Jordan , W. C. Coates , S. Clarke , et al., “The Uphill Battle of Performing Education Scholarship: Barriers Educators and Education Researchers Face,” Western Journal of Emergency Medicine 19, no. 3 (2018): 619–629. 10.5811/westjem.2018.1.36752 PMC5942034 29760865

[13] G. Carmelli , M. Cassara , D. T. Miller , et al., “From Submission to Publication: A Practical Guide to Peer Review Publication for Medical Educators,” AEM Education and Training 9, no. Suppl 1 (2025): S88–S100. 10.1002/aet2.70023 PMC12038857 40308864

[14] Biosemantics Group , “Journal/Author Name Estimator (JANE),” accessed June 24, 2026, https://jane.biosemantics.org.

[15] A. Berry , P. Herring , and J. M. Spak , Annotated Bibliography of Journals for Educational Scholarship (AAMC Medical Education Scholarship, Research and Evaluation (MESRE) Section, 2024), accessed June 24, 2026, https://www.aamc.org/media/38166/download?attachment.

[16] L. S. Murphy , C. K. Kraus , S. Lotfipour , M. Gottlieb , J. R. Langabeer, 2nd , and M. I. Langdorf , “Measuring Scholarly Productivity: A Primer for Junior Faculty. Part III: Understanding Publication Metrics,” Western Journal of Emergency Medicine 19, no. 6 (2018): 1003–1011. 10.5811/westjem.2018.9.38213 PMC6225941 30429933

[17] S. Moore , “Submitting a Manuscript to a Scientific Journal,” Respiratory Care 68, no. 9 (2023): 1314–1319. 10.4187/respcare.11054 PMC10468169 37253607

[18] M. Sharma , A. Sarin , P. Gupta , S. Sachdeva , and A. V. Desai , “Journal Impact Factor: Its Use, Significance and Limitations,” World Journal of Nuclear Medicine 13 (2014): 146. 10.4103/1450-1147.139151 PMC4150161 25191134

[19] T. M. Chan , B. Thoma , A. K. Hall , et al., “CAEP 2016 Academic Symposium: A Writer's Guide to Key Steps in Producing Quality Medical Education Scholarship,” CJEM 19, no. S1 (2017): S9–S15. 10.1017/cem.2017.30 28508740

[20] S. Matsubara and D. Matsubara , “A Checklist Confirming Whether a Manuscript for Submission Adheres to the Fundamentals of Academic Writing: A Proposal,” JMA Journal 7, no. 2 (2024): 276–278. 10.31662/jmaj.2023-0201 PMC11074514 38721070

[21] M. Gottlieb , J. A. Kline , A. J. Schneider , and W. C. Coates , “ChatGPT and Conversational Artificial Intelligence: Friend, Foe, or Future of Research?,” American Journal of Emergency Medicine 70 (2023): 81–83. 10.1016/j.ajem.2023.05.018 37229893

[22] H. Hachfi , M. Kechida , R. Kaddoussi , et al., “Writing an Effective and Succinct Cover Letter: A Practical Guide,” La Tunisie Médicale 102, no. 12 (2024): 988–994. 10.62438/tunismed.v102i12.5438 PMC11770805 39748680

[23] Z. Bahadoran , P. Mirmiran , K. Kashfi , and A. Ghasemi , “Scientific Publishing in Biomedicine: How to Write a Cover Letter?,” International Journal of Endocrinology and Metabolism 19, no. 3 (2021): e115242. 10.5812/ijem.115242 PMC8453652 34567139

[24] K. W. Eva , “The Reviewer Is Always Right: Peer Review of Research in Medical Education,” Medical Education 43, no. 1 (2009): 2–4. 10.1111/j.1365-2923.2008.03243.x 19140991

[25] D. K. Nakayama , “Turning a Rejection Into an Accepted Manuscript: How to Rework a Rejected Manuscript,” American Surgeon 92, no. 4 (2026): 1059–1061. 10.1177/00031348261416454 41517934

[26] R. Smith , “Peer Review: A Flawed Process at the Heart of Science and Journals,” Journal of the Royal Society of Medicine 99, no. 4 (2006): 178–182. 10.1258/jrsm.99.4.178 PMC1420798 16574968

[27] S. V. Kotsis and K. C. Chung , “Manuscript Rejection: How to Submit a Revision and Tips on Being a Good Peer Reviewer,” Plastic and Reconstructive Surgery 133, no. 4 (2014): 958–964. 10.1097/PRS.0000000000000002 PMC3970178 24675196

[28] N. S. Trueger , E. Aly , S. Haneuse , E. Huang , and M. Berkwits , “Randomized Clinical Trial Visual Abstract Display and Social Media‐Driven Website Traffic,” JAMA 330, no. 16 (2023): 1583–1585. 10.1001/jama.2023.16839 PMC10599120 37773505

[29] M. Gottlieb , A. M. Ibrahim , L. J. Martin , Y. Yilmaz , and T. M. Chan , “Educator's Blueprint: A How‐To Guide for Creating a High‐Quality Infographic,” AEM Education and Training 6, no. 4 (2022): e10793. 10.1002/aet2.10793 PMC9411917 36034885

[30] M. Gottlieb , T. M. Chan , S. O. Clarke , et al., “Emergency Medicine Education Research Since the 2012 Consensus Conference: How Far Have We Come and What's Next?,” AEM Education and Training 4, no. Suppl 1 (2019): S57–S66. 10.1002/aet2.10404 PMC7011414 32072108

[31] G. Comp , S. Dyer , and M. Gottlieb , “Is TikTok the Next Social Media Frontier for Medicine?,” AEM Education and Training 5, no. 3 (2020): 10532, 10.1002/aet2.10532. PMC8155692 34095694

[32] M. Gottlieb and S. Dyer , “Information and Disinformation: Social Media in the COVID‐19 Crisis,” Academic Emergency Medicine 27, no. 7 (2020): 640–641. 10.1111/acem.14036 PMC7300599 32474977

[33] M. A. Gisondi , R. Barber , J. S. Faust , et al., “A Deadly Infodemic: Social Media and the Power of COVID‐19 Misinformation,” Journal of Medical Internet Research 24, no. 2 (2022): e35552. 10.2196/35552 PMC8812140 35007204

[34] S. Schrager and E. Sadowski , “Getting More Done: Strategies to Increase Scholarly Productivity,” Journal of Graduate Medical Education 8, no. 1 (2016): 10–13. 10.4300/JGME-D-15-00165.1 PMC4763375 26913095

[35] M. Gottlieb , H. Caretta‐Weyer , T. M. Chan , and S. Humphrey‐Murto , “Educator's Blueprint: A Primer on Consensus Methods in Medical Education Research,” AEM Education and Training 7, no. 4 (2023): e10891. 10.1002/aet2.10891 PMC10336022 37448627

