# Supplementary material for: Educator's Blueprint: Seven Strategies for Turning Peer Review Into Scholarly Growth
Source: AEM Educ Train. 2026 Oct 6;10(5):e70265. doi: 10.1002/aet2.70265 (PMC13642983; doi:10.1002/aet2.70265)
Supplement: Supplementary file 1 [file AET2-10-e70265-s001.docx]

SUPPLEMENTAL FIGURE 1a: Suggested journals for the current article using title only

SUPPLEMENTAL Figure 1b: Suggested journals for the current article using the abstract
